# Perioperative Inflammatory Cytokines in Parkinson’s Disease

**DOI:** 10.3390/biom16020261

**Published:** 2026-02-05

**Authors:** Jong-Woan Kim, Seung-ah Yoo, Yemi Choi, Gi Heon Jeong, Jaeseung Lee, Jin Joo

**Affiliations:** 1 Department of Anesthesiology and Pain Medicine, Seoul St. Mary’s Hospital, College of Medicine, The Catholic University of Korea, Seoul 06591, Republic of Korea; jwjw12@naver.com (J.-W.K.);; 2 Department of Medical Life Sciences, College of Medicine, The Catholic University of Korea, Seoul 06591, Republic of Korea; youcap78@catholic.ac.kr (S.-a.Y.);

**Keywords:** Parkinson’s disease, neuroinflammation, cytokines, perioperative period, IL-6, IL-8, VEGF

## Abstract

**Background**: Neuroinflammation is increasingly recognized as an important contributor to Parkinson’s disease (PD), yet perioperative immune responses in this population remain incompletely characterized. This study investigated perioperative cytokine dynamics in patients with PD compared with healthy controls (HCs) undergoing orthopedic surgery under general anesthesia. **Methods**: In this prospective pilot observational study, 50 patients scheduled for lower limb orthopedic surgery were enrolled (25 PD patients, 25 HCs). Serum cytokines (IL-6, IL-8, VEGF, MCP-1, HMGB1, S100B, and PARK7) were measured immediately after anesthesia induction (PRE) and 24 h postoperatively (POST). Between-group comparisons were performed using independent *t*-tests, and within-group perioperative changes were assessed using paired *t*-tests. Absolute (Δ = POST − PRE) and relative perioperative changes were analyzed. **Results**: IL-6 increased significantly after surgery in both groups, with no significant differences in absolute or relative perioperative changes between the PD and HC group. IL-8 concentrations were numerically higher in PD patients at both time points, but perioperative changes did not differ significantly between groups. VEGF decreased modestly within the PD group, whereas no significant change was observed in HCs; however, between-group differences in perioperative VEGF changes were not significant. S100B and PARK7 increased postoperatively in HCs but not in PD patients, while MCP-1 and HMGB1 showed no significant perioperative changes. **Conclusions**: In this pilot study, perioperative cytokine responses in patients with PD were largely comparable to those in HCs. Despite evidence of chronic low-grade inflammation in the PD group, no disease-specific amplification of acute perioperative inflammatory responses was observed. These findings suggest that perioperative immune activation in PD may be selective rather than global.

## 1. Introduction

Parkinson’s disease (PD) is the second most prevalent neurodegenerative disorder and the most rapidly increasing in incidence, affecting approximately 1% of individuals over the age of 65 worldwide [1,2,3]. Clinically, PD is characterized by motor symptoms such as resting tremor and dyskinesia, as well as a wide range of non-motor manifestations, including gastrointestinal dysfunction, cognitive decline, depression, and pain. Pathologically, PD is defined by the progressive degeneration of dopaminergic neurons in the substantia nigra and the accumulation of intraneuronal Lewy bodies [3].

Despite extensive research, the precise mechanisms underlying PD pathogenesis remain incompletely understood. Growing evidence highlights neuroinflammation as a central contributor to PD progression [1,4,5,6]. Microglial activation and peripheral–central immune crosstalk are increasingly recognized as key mechanisms linking systemic inflammation to neuronal vulnerability [1,7]. These immune processes are reflected in measurable changes in circulating and cerebrospinal fluid biomarkers. In particular, proinflammatory cytokines such as interleukin (IL)-6, IL-8, tumor necrosis factor-α (TNF-α), interferon-γ (IFN-γ), and monocyte chemoattractant protein-1 (MCP-1) have been implicated in neurodegenerative cascades, while molecules including vascular endothelial growth factor (VEGF), high-mobility group box 1 (HMGB1), S100 calcium-binding protein B (S100B), and Parkinson disease protein 7 (PARK7/DJ-1) have been proposed as indicators of neuroinflammation, oxidative stress, or compensatory neuroprotective mechanisms [6,8,9,10,11,12].

Surgical stress and general anesthesia represent clinically relevant triggers of systemic immune activation. Aseptic tissue injury provokes cytokine release and may disrupt the integrity of the blood–brain barrier, thereby amplifying neuroinflammatory responses [13,14,15]. Such perioperative immune alterations are of particular concern in PD, where patients may be predisposed to exaggerated inflammatory responses and are clinically vulnerable to postoperative neurocognitive disorders [7,16].

Although numerous studies have explored cytokine alterations in PD and in the context of perioperative neuroinflammation separately, little is known about how cytokine dynamics differ perioperatively between patients with PD and otherwise healthy individuals. Therefore, the present study was designed to investigate perioperative changes in circulating cytokines and related biomarkers in patients with PD compared with age- and sex-matched healthy controls undergoing orthopedic surgery under general anesthesia.

## 2. Materials and Methods

### 2.1. Study Population and Ethical Approval

Written and verbal informed consent was obtained from all participants prior to enrollment. This prospective observational study enrolled patients with PD (PD group) aged 40–80 years who were undergoing lower limb orthopedic surgery under general anesthesia, together with age- and sex-matched healthy controls (HC group) who were scheduled for the same procedures, between January 2023 and August 2025. Exclusion criteria were as follows: a history of myocardial infarction or coronary artery disease (other than hypertension-related or diabetic heart disease), chronic pulmonary conditions such as asthma or chronic obstructive pulmonary disease (COPD), abnormal liver function, defined as elevated AST or ALT above the normal range, and any history of hypersensitivity to inhalational anesthetics or propofol.

### 2.2. Anesthetic Management

All patients fasted from food and fluids for at least 8 h before surgery. Upon arrival in the operating room, standard monitoring was applied, including electrocardiography (ECG), non-invasive blood pressure (NIBP), pulse oximetry, and bispectral index (BIS). General anesthesia was induced with intravenous propofol (1–2 mg/kg) and rocuronium (0.6–1 mg/kg), followed by tracheal intubation after 1–2 min. Anesthesia was maintained with sevoflurane (1–2 vol%), remifentanil (0.01–0.2 µg/kg/min), and a mixture of oxygen and air (FiO_2_ 0.4). The depth of anesthesia was adjusted to maintain a BIS between 40 and 60, and hemodynamic parameters (blood pressure and heart rate) were controlled within ±20% of baseline values. The first blood sample (PRE) was collected immediately after induction of anesthesia. At the end of surgery, anesthetic agents were discontinued, and sugammadex was administered to reverse neuromuscular blockade. Extubation was performed once adequate spontaneous ventilation was confirmed, and patients were transferred to the recovery room. A second blood sample (POST) was obtained 24 h after surgery from peripheral venous access.

### 2.3. Cytokine Analysis

Peripheral venous blood was collected from each participant into serum separator tubes and centrifuged at 1000 rpm for 10 min at room temperature. The supernatant serum was then aliquoted into sterile 1.5 mL microcentrifuge tubes (500 µL per tube) and stored at −80 °C until analysis. Circulating cytokine concentrations were quantified using commercially available sandwich enzyme-linked immunosorbent assay (ELISA) kits, following the manufacturers’ instructions. The following analytes were assessed: IL-6, IL-8, MCP-1, TNF-α, IFN-γ, VEGF, S100B, and PARK7 (all from R&D Systems, Minneapolis, MN, USA), as well as HMGB1 (Invitrogen, Carlsbad, CA, USA).

### 2.4. Data Collection and Statistical Analysis

Since this prospective observational study was designed as an exploratory pilot investigation, no formal a priori sample size or power calculation was performed. Analyses were conducted using SPSS Statistics (version 29, IBM Corp., Armonk, NY, USA). Demographic data were compared using the χ^2^ test and *t*-test as appropriate. Continuous variables are presented as mean ± standard deviation (SD). Between-group comparisons (PD group vs. HC group) at each time point were conducted using independent two-sample *t*-tests. Within-group perioperative changes (PRE vs. POST) were assessed using paired *t*-tests. Absolute perioperative changes (Δ = POST − PRE) and relative changes ((POST − PRE)/PRE × 100) were calculated and compared between groups using independent *t*-tests. Given the exploratory nature of this prospective pilot study, no formal correction for multiple comparisons was applied. A two-sided *p*-value < 0.05 was considered statistically significant.

## 3. Results

**Figure 1:**
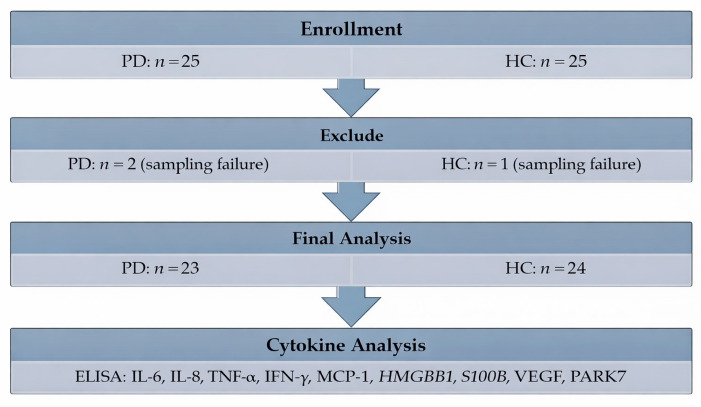
Study flow diagram. PD: patients with Parkinson’s disease; HC: healthy control; IL: interleukin; TNF: tumor necrosis factor; IFN: interferon; MCP: monocyte chemoattractant protein; HMGB: high-mobility group box 1; S100B: S100 calcium-binding protein B; VEGF: vascular endothelial growth factor; PARK: Parkinson disease protein.

**Figure 2:**
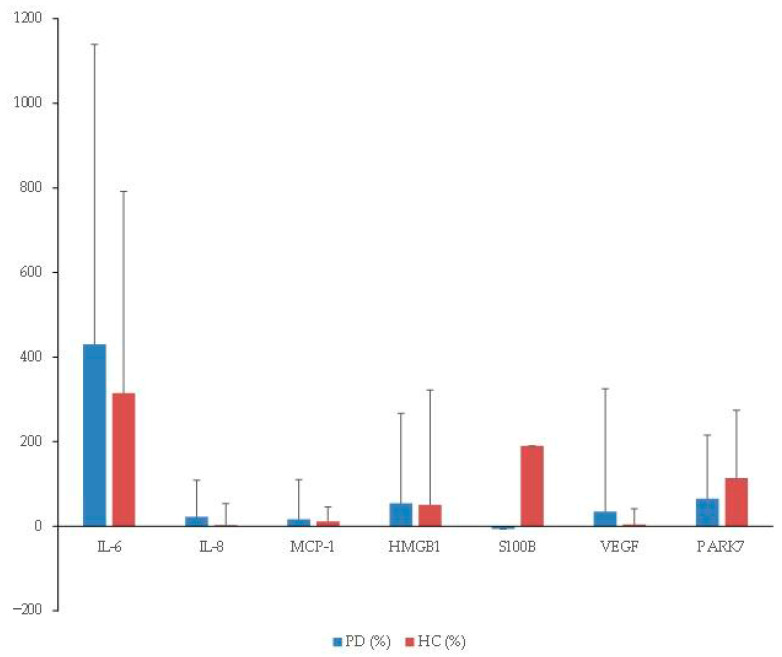
Relative perioperative cytokine changes. Relative changes were calculated as [(Postoperative − Preoperative)/Preoperative × 100] and are expressed as percentages. Data are presented as mean ± standard deviation (SD). Groups were compared using independent *t*-tests (PD, *n* = 23; HC, *n* = 24). No statistically significant differences in relative changes were observed between the two groups. PD: patients with Parkinson’s disease; HC: healthy control; IL: interleukin; MCP: monocyte chemoattractant protein; HMGB: high-mobility group box 1; S100B: S100 calcium-binding protein B; VEGF: vascular endothelial growth factor; PARK: Parkinson disease protein.

A total of 50 patients (25 in each group) were enrolled. Three were excluded from the final analysis due to loss to follow-up (two in the PD group and one in the HC group) (Figure 1). Demographic and perioperative clinical data are summarized in Table 1. TNF-α and IFN-γ were detectable in fewer than 20% of samples; therefore, statistical analysis was not performed for these cytokines. Substantial inter-individual variability was observed across cytokines, and no relative perioperative changes differed significantly between patients with PD and the HCs (Figure 2).

For IL-6, PRE concentrations were numerically higher in PD patients compared with HCs, although this difference did not reach statistical significance (*p* = 0.091). POST IL-6 was significantly elevated in both groups (PD: *p* = 0.041; HC: *p* < 0.001). Although the absolute perioperative increase (Δ) was greater in PD patients than in HCs (29.9 ± 62.9 vs. 8.6 ± 9.1 pg/mL), this between-group difference did not reach statistical significance (*p* = 0.139). Relative changes were also comparable between groups (*p* = 0.732) (Table 2, Figure 2).

While the increase in PRE IL-8 did not reach statistical significance (*p* = 0.056), a numerical trend toward higher levels in the PD group was observed. POST IL-8 concentrations remained elevated compared with HCs. However, neither absolute nor relative perioperative changes (Δ) showed significant between-group differences (Table 2, Figure 2).

For VEGF, both groups exhibited modest perioperative decreases. A significant reduction was observed within the PD group (*p* = 0.034), whereas no significant change was detected in the HC group. However, neither the absolute perioperative change (Δ) nor the relative perioperative change differed significantly between groups (Table 2, Figure 2).

S100B and PARK7 showed significant postoperative increases within the HC group. However, absolute and relative perioperative changes (Δ) were not significantly different between the PD and HC group (Table 2, Figure 2). MCP-1 and HMGB1 did not show significant perioperative differences within or between groups (Table 2).

## 4. Discussion

Although the precise mechanisms underlying PD pathogenesis remain unclear, increasing evidence suggests that neuroinflammation plays a pivotal role in the pathogenesis and progression of PD [1,4,5,6,7,16,17]. Microglial activation and peripheral–central immune crosstalk are increasingly recognized as key mechanisms contributing to neuronal vulnerability in the substantia nigra and beyond [1,7]. These immunological processes are reflected in measurable changes in circulating and cerebrospinal fluid biomarkers, particularly proinflammatory cytokines such as IL-6, IL-1β, TNF-α, and IL-8, which have been associated with PD severity and progression in previous studies [6,8,9,10,11,12]. Importantly, recent reviews highlight that targeting neuroinflammatory pathways, including cytokine signaling, may represent a promising strategy for disease modification in PD [7,16]. Together, these findings support the concept that neuroinflammation, mediated in part by cytokine dysregulation, is a critical contributor to PD pathophysiology and a potential avenue for therapeutic intervention.

In this prospective observational study, we investigated perioperative cytokine dynamics in PD patients compared with age- and sex-matched healthy controls undergoing orthopedic surgery under general anesthesia. Our principal findings were as follows: (1) IL-6 showed significant perioperative increases in both groups, with numerically greater absolute changes in PD patients, although between-group differences were not statistically significant; (2) IL-8 was consistently higher in PD patients both at baseline and postoperatively, but perioperative fluctuations did not differ significantly between groups; (3) VEGF decreased significantly within the PD group but not in controls, yet between-group comparisons of perioperative change did not reach significance; and (4) S100B and PARK7 increased significantly postoperatively in healthy controls, although no significant differences in Δ values were observed between the PD and HC group. MCP-1 and HMGB1 demonstrated no perioperative alterations.

IL-6 is a multifunctional cytokine that is secreted mainly by neurons and glial cells, which plays a crucial role in the development and differentiation of neurons [18]. In the present study, although the absolute perioperative increase in IL-6 was numerically greater in the PD group, the between-group difference in Δ values did not reach statistical significance. Relative perioperative changes were also comparable between groups. This pattern supports IL-6 as a sensitive marker of systemic inflammatory responses to surgical stress and anesthesia rather than a PD-specific effect [13,14,15]. Elevated IL-6 has also been frequently reported in PD and linked to both motor and non-motor symptom severity [4,5,8,10,11,17], although not all studies have shown consistent results [6,19]. Importantly, baseline IL-6 measurements in the present study were obtained immediately after anesthesia induction and did not differ significantly between groups; therefore, the present data do not support the presence of a definitive pre-existing inflammatory state that is attributable to PD. General anesthesia itself is known to modulate immune signaling, including cytokine release, even during the early induction phase. Consequently, the observed PRE cytokine profiles likely reflect a combination of disease-related susceptibility and peri-anesthetic immune modulation. Moreover, the absence of significant between-group differences in perioperative IL-6 changes precludes clinical inference regarding disease-specific vulnerability to perioperative neurological complications. Larger, outcome-linked studies incorporating true pre-anesthetic baseline measurements will be required to determine whether PD confers a distinct perioperative IL-6 response.

IL-8 is a chemokine, which is produced by macrophages in response to proinflammatory mediators [20]. In the present study, IL-8 exhibited a distinct pattern compared with other cytokines. PRE IL-8 concentrations showed a trend toward higher values in patients with PD compared with the HCs, and POST levels remained numerically elevated; however, perioperative changes did not differ significantly between groups. These findings suggest that IL-8 may reflect a chronic low-grade immune activation associated with PD rather than an acute response to surgical stress. Nevertheless, these findings should be interpreted cautiously given the modest sample size and marked inter-individual variability, and no definitive conclusions regarding PD-specific perioperative IL-8 dynamics can be drawn. Previous studies have demonstrated increased IL-8 secretion from peripheral immune cells in prodromal PD [6], and serum elevations have been associated with disease severity [11], although meta-analyses have yielded inconsistent findings, with some studies reporting elevated IL-8 in PD and others showing no significant differences [10,21]. Our data supports the hypothesis that IL-8 may be more relevant as a marker of sustained neuroimmune dysregulation than as a perioperative biomarker of acute stress responses. Further longitudinal studies are warranted to determine whether IL-8 contributes more to chronic neuroinflammation than to acute perioperative immune activation.

Although PD has been associated with chronic low-grade inflammation [4,7,17], the present data do not demonstrate a disease-specific amplification of acute perioperative inflammatory responses. In particular, perioperative changes in IL-6 and IL-8 were not significantly different between patients with PD and HCs. These findings indicate that a chronic inflammatory state in PD does not necessarily result in an exaggerated systemic immune response to surgical stress.

VEGF demonstrated a modest postoperative decrease in patients with PD, whereas no significant change was observed in HCs. However, neither absolute nor relative perioperative VEGF changes differed significantly between groups. Although experimental studies have shown that exogenous VEGF exerts neuroprotective effects in PD models by promoting angiogenesis and dopaminergic neuron survival [22,23,24,25], the present findings do not provide evidence of a PD-specific perioperative VEGF response. The absence of a perioperative increase in VEGF among patients with PD should therefore be interpreted cautiously and may reflect nonspecific effects of surgical or anesthetic stress rather than impaired neurovascular resilience that is unique to the disease. In addition, discrepancies with prior reports of increased cerebrospinal fluid VEGF in PD [23] may be attributable to fundamental differences between serum and cerebrospinal fluid measurements, as circulating VEGF levels may not accurately represent central nervous system-specific signaling. Consequently, the clinical and mechanistic implications of perioperative VEGF dynamics remain uncertain and warrant further investigation in larger studies incorporating cerebrospinal fluid biomarkers and clinical outcome measures.

S100B and PARK7 increased significantly in HCs but not in patients with PD. Rather than indicating reduced perioperative vulnerability in PD, this pattern may reflect differences in baseline regulation or dynamic responsiveness of glial and oxidative stress-related pathways [12,26,27]. In HCs, postoperative increases in S100B and PARK7 may represent an intact, inducible response to surgical stress, whereas in PD patients, these pathways may already be chronically altered, resulting in attenuated or non-detectable perioperative changes. These findings challenge the assumption that patients with Parkinson’s disease uniformly exhibit exaggerated perioperative neuroinflammatory responses. MCP-1 and HMGB1 showed no significant perioperative changes. Prior studies have associated MCP-1 with PD progression [28,29] and HMGB1 with postoperative cognitive dysfunction and cognitive decline in PD [30,31]. The absence of perioperative alterations in our study may be attributable to methodological factors, including small sample size, limited sampling time points, and the use of serum rather than CSF.

These negative results are clinically informative, as they challenge the assumption that patients with Parkinson’s disease uniformly exhibit exaggerated perioperative neuroinflammatory responses. The lack of differential perioperative responses in these biomarkers suggests that perioperative immune activation in Parkinson’s disease may be selective rather than global. While certain cytokines such as IL-6, IL-8, and VEGF demonstrated distinct patterns, others remained comparable to healthy controls, underscoring the heterogeneity of immune dysregulation in Parkinson’s disease.

This study has several limitations. First, the modest sample size may have limited the statistical power, and cytokine levels were only measured at two perioperative time points, potentially missing transient or delayed responses. Consequently, the absence of statistically significant Δ differences for key cytokines such as IL-6 and VEGF precludes definitive conclusions regarding PD-specific perioperative inflammatory responses. Second, cytokine measurements were restricted to serum samples. PD is fundamentally a central nervous system disorder, and peripheral cytokine levels may not accurately reflect neuroinflammatory processes occurring within the brain. Although peripheral inflammatory markers may provide indirect insight into systemic immune activation and peripheral–central immune crosstalk, they cannot be considered definitive surrogates of central neuroinflammation [7,15,23]. Third, perioperative neurological and cognitive outcomes and correlations between cytokine levels and clinical disease characteristics were not assessed; therefore, the clinical significance of the observed perioperative cytokine patterns cannot be directly determined. Lastly, the statistical approach used in the present study warrants cautious interpretation. Multiple cytokines were examined without adjustment for multiple comparisons, increasing the possibility of type I error. In addition, substantial non-normal distributions may also increase the risk of type II error, particularly in a modestly sized cohort. Despite these limitations, this study has important strengths. To our knowledge, it is the first investigation to directly compare perioperative cytokine responses between patients with PD and HCs undergoing surgery under general anesthesia. As an exploratory pilot study, the present study intended to provide preliminary effect size estimates to inform the power calculations of future, larger-scale prospective trials. It demonstrates feasibility and provides an initial characterization of perioperative immune dynamics in this population. Importantly, the presence of both positive and negative findings suggests that perioperative immune alterations in PD may be selective rather than global. These results provide a need for larger, longitudinal studies incorporating cerebrospinal fluid biomarkers and standardized clinical outcome assessments to clarify the perioperative neuroinflammatory response in PD.

## 5. Conclusions

In conclusion, this prospective pilot study provides the first direct comparison of perioperative cytokine responses between patients with PD and HCs undergoing orthopedic surgery under general anesthesia. Despite evidence of chronic low-grade inflammation in Parkinson’s disease, perioperative changes in key cytokines, including IL-6 and IL-8, were not significantly different between groups, and no disease-specific amplification of acute inflammatory responses was observed. These findings suggest that perioperative immune activation in Parkinson’s disease may be selective rather than global. Larger studies incorporating central biomarkers and clinical outcome measures are needed to clarify the clinical relevance of perioperative inflammatory responses in this population.

## Figures and Tables

**Table 1:** Patient and clinical data.

	PD Group (*n* = 23)	HC Group (*n* = 24)	*p*-Value
Age (yr)	67.5 ± 8.7	66.5 ± 6.6	0.654
Gender (F/M)	9/14	11/13	0.865
Height (cm)	160.4 ± 10.0	161.7 ± 7.9	0.619
Weight (kg)	65.2 ± 13.3	64.5 ± 9.8	0.823
Duration of surgery (min)	113.5 ± 45.5	101.7 ± 47.4	0.597
Duration of anesthesia (min)	141.7 ± 53.3	134.4 ± 47.6	0.757
Fluid administered (mL)	394.8 ± 93.8	336.7 ± 98.4	0.778
Blood loss (mL)	140.2 ± 86.4	168.3 ± 85.4	0.531
Propofol administered (mg)	101.3 ± 23.6	111.7 ± 22.2	0.129
Remifentanil administered (mL)	0.5 ± 0.3	0.5 ± 0.2	0.768
PD yrs (PD group only)	9.9 ± 6.5		

PD: patients with Parkinson’s disease; HC: healthy control.

**Table 2:** Perioperative cytokine changes.

	Group	PRE	POST	Delta (PRE − POST)	*p*-Value (PRE vs. POST)	*p*-Value (PRE, PD vs. HC	*p*-Value (Delta, PD vs. HC)
IL-6 (pg/mL)	PD	19.8 ± 7.5	50.6 ± 15.6	29.9 ± 12.9	0.041	0.091	0.139
	HC	6.4 ± 11.9	15.1 ± 19.0	8.6 ± 9.1	<0.001		
IL-8 (pg/mL)	PD	17.5 ± 18.0	14.1 ± 10.7	−3.4 ± 11.1	0.156	0.056	0.2556
	HC	9.4 ± 7.2	8.8 ± 6.1	−0.6 ± 2.8	0.290		
MCP-1 (pg/mL)	PD	454.2 ± 659.2	429.8 ± 471.7	−24.4 ± 264.1	0.662	0.186	0.414
	HC	265.8 ± 66.3	259.9 ± 107.2	24.2 ± 98.6	0.242		
HMGB1 (pg/mL)	PD	3055.5 ± 2084.6	3222.0 ± 2502.3	166.5 ± 2883.0	0.784	0.192	0.910
	HC	4227.2 ± 3714.7	4310.7 ± 3601.1	83.5 ± 2028.0	0.842		
S100B (pg/mL)	PD	0.7 ± 3.3	9.5 ± 33.0	8.8 ± 33.0	0.216	0.625	0.132
	HC	1.5 ± 7.5	28.7 ± 45.6	27.2 ± 43.5	0.006		
VEGF (pg/mL)	PD	116.3 ± 113.9	90.7 ± 108.9	−25.7 ± 54.3	0.034	0.393	0.108
	HC	92.3 ± 71.6	87.2 ± 66.4	−5.1 ± 34.8	0.483		
PARK7 (ng/mL)	PD	20.9 ± 51.8	9.1 ± 7.2	−11.8 ± 51.6	0.256	0.155	0.151
	HC	5.0 ± 2.7	9.3 ± 5.9	4.3 ± 5.5	<0.001		

PD: patients with Parkinson’s disease; HC: healthy control; IL: interleukin; MCP: monocyte chemoattractant protein; HMGB: high-mobility group box 1; S100B: S100 calcium-binding protein B; VEGF: vascular endothelial growth factor; PARK: Parkinson disease protein; PRE: immediately after anesthesia induction; POST: 24 h after surgery.

## Data Availability

The data presented in this study are available on request from the corresponding author. The data are not publicly available due to concerns over protecting the personal privacy of the participants, as well as because the author plans to continue using this dataset for ongoing and future related research, these data have not been made public for the time being.

## Abbreviations

The following abbreviations are used in this manuscript:

PD	Parkinson’s disease
IL	Interleukin
TNF-α	Tumor necrosis factor-α
IFN-γ	Interferon-γ
MCP-1	Monocyte chemoattractant protein-1
VEGF	Vascular endothelial growth factor
HMGB1	High-mobility group box 1
S100B	S100 calcium-binding protein B
PARK7	Parkinson disease protein 7
HC	Healthy control
PRE	Immediately after anesthesia induction
POST	24 h after surgery

## References

[1] Mogensen F.L.-H., Seibler P., Grünewald A., Michelucci A. (2025). Microglial dynamics and neuroinflammation in prodromal and early Parkinson’s disease. J. Neuroinflamm..

[2] Dorsey E.R., Bloem B.R. (2018). The Parkinson Pandemic—A Call to Action. JAMA Neurol..

[3] Bloem B.R., Okun M.S., Klein C. (2021). Parkinson’s disease. Lancet.

[4] Chen X., Feng W., Ou R., Liu J., Yang J., Fu J., Cao B., Chen Y., Wei Q., Shang H. (2021). Evidence for Peripheral Immune Activation in Parkinson’s Disease. Front. Aging Neurosci..

[5] Greenland J.C., Holbrook J., Kahanawita L., Camacho M., Fryer T.D., Hong Y.T., Williams-Gray C.H. (2025). Peripheral–central immune crosstalk in Parkinson’s disease and its association with clinical severity. Brain Behav. Immun..

[6] Mark J.R., Titus A.M., Staley H.A., Alvarez S., Mahn S., McFarland N.R., Wallings R.L., Tansey M.G. (2025). Peripheral immune cell response to stimulation stratifies Parkinson’s disease progression from prodromal to clinical stages. Commun. Biol..

[7] Tansey M.G., Wallings R.L., Houser M.C., Herrick M.K., Keating C.E., Joers V. (2022). Inflammation and immune dysfunction in Parkinson disease. Nat. Rev. Immunol..

[8] Zimmermann M., Brockmann K. (2022). Blood and Cerebrospinal Fluid Biomarkers of Inflammation in Parkinson’s Disease. J. Park. Dis..

[9] Ma Z.L., Wang Z.L., Zhang F.Y., Liu H.X., Mao L.H., Yuan L. (2024). Biomarkers of Parkinson’s Disease: From Basic Research to Clinical Practice. Aging Dis..

[10] Williams-Gray C.H., Wijeyekoon R.S., Yarnall A.J., Lawson R.A., Breen D.P., Evans J.R., Cummins G.A., Duncan G.W., Khoo T.K., Burn D.J. (2016). Serum immune markers and disease progression in an incident Parkinson’s disease cohort (ICICLE-PD). Mov. Disord..

[11] Fu J., Chen S., Liu J., Yang J., Ou R., Zhang L., Chen X., Shang H. (2023). Serum inflammatory cytokines levels and the correlation analyses in Parkinson’s disease. Front. Cell Dev. Biol..

[12] Papuć E., Rejdak K. (2020). Increased Cerebrospinal Fluid S100B and NSE Reflect Neuronal and Glial Damage in Parkinson’s Disease. Front. Aging Neurosci..

[13] Liu Y., Yang W., Xue J., Chen J., Liu S., Zhang S., Zhang X., Gu X., Dong Y., Qiu P. (2023). Neuroinflammation: The central enabler of postoperative cognitive dysfunction. Biomed. Pharmacother..

[14] Li T., Lu Z., Qin T., Liu L., Zhang J. (2025). Pathomechanism of postoperative delirium: Systemic inflammatory response and neuroinflammation following anesthesia/surgery. Neuroscience.

[15] Yang T., Velagapudi R., Terrando N. (2020). Neuroinflammation after surgery: From mechanisms to therapeutic targets. Nat. Immunol..

[16] Grotemeyer A., McFleder R.L., Wu J., Wischhusen J., Ip C.W. (2022). Neuroinflammation in Parkinson’s Disease—Putative Pathomechanisms and Targets for Disease-Modification. Front. Immunol..

[17] Qu Y., Li J., Qin Q., Wang D., Zhao J., An K., Mao Z., Min Z., Xiong Y., Li J. (2023). A systematic review and meta-analysis of inflammatory biomarkers in Parkinson’s disease. npj Park. Dis..

[18] Borsche M., König I.R., Delcambre S., Petrucci S., Balck A., Brüggemann N., Zimprich A., Wasner K., Pereira S.L., Avenali M. (2020). Mitochondrial damage-associated inflammation highlights biomarkers in PRKN/PINK1 parkinsonism. Brain.

[19] Álvarez-Luquín D.D., Arce-Sillas A., Leyva-Hernández J., Sevilla-Reyes E., Boll M.C., Montes-Moratilla E., Vivas-Almazán V., Pérez-Correa C., Rodríguez-Ortiz U., Espinoza-Cárdenas R. (2019). Regulatory impairment in untreated Parkinson’s disease is not restricted to Tregs: Other regulatory populations are also involved. J. Neuroinflamm..

[20] Righi D., Manco C., Pardini M., Stufano A., Schino V., Pelagotti V., Massa F., De Stefano N., Plantone D. (2025). Investigating interleukin-8 in Alzheimer’s disease: A comprehensive review. J. Alzheimer’s Dis..

[21] Qin X.Y., Zhang S.P., Cao C., Loh Y.P., Cheng Y. (2016). Aberrations in Peripheral Inflammatory Cytokine Levels in Parkinson Disease: A Systematic Review and Meta-analysis. JAMA Neurol..

[22] Shim J.W., Madsen J.R. (2018). VEGF Signaling in Neurological Disorders. Int. J. Mol. Sci..

[23] Janelidze S., Lindqvist D., Francardo V., Hall S., Zetterberg H., Blennow K., Adler C.H., Beach T.G., Serrano G.E., van Westen D. (2015). Increased CSF biomarkers of angiogenesis in Parkinson disease. Neurology.

[24] Yasuhara T., Shingo T., Kobayashi K., Takeuchi A., Yano A., Muraoka K., Matsui T., Miyoshi Y., Hamada H., Date I. (2004). Neuroprotective effects of vascular endothelial growth factor (VEGF) upon dopaminergic neurons in a rat model of Parkinson’s disease. Eur. J. Neurosci..

[25] Falk T., Zhang S., Sherman S.J. (2009). Vascular endothelial growth factor B (VEGF-B) is up-regulated and exogenous VEGF-B is neuroprotective in a culture model of Parkinson’s disease. Mol. Neurodegener..

[26] Hong Z., Shi M., Chung K.A., Quinn J.F., Peskind E.R., Galasko D., Jankovic J., Zabetian C.P., Leverenz J.B., Baird G. (2010). DJ-1 and α-synuclein in human cerebrospinal fluid as biomarkers of Parkinson’s disease. Brain.

[27] Ozturk N.K., Kavakli A.S., Arslan U., Aykal G., Savas M. (2020). S100B level and cognitive dysfunction after robotic-assisted laparoscopic radical prostatectomy procedures: A prospective observational study. Braz. J. Anesthesiol..

[28] Shen R., Lin S., He L., Zhu X., Zhou Z., Chen S., Wang Y., Ding J. (2019). Association of Two Polymorphisms in CCL2 with Parkinson’s Disease: A Case-Control Study. Front. Neurol..

[29] Santaella A., Kuiperij H.B., van Rumund A., Esselink R.A.J., van Gool A.J., Bloem B.R., Verbeek M.M. (2020). Cerebrospinal fluid monocyte chemoattractant protein 1 correlates with progression of Parkinson’s disease. npj Park. Dis..

[30] Mao D., Zheng Y., Xu F., Han X., Zhao H. (2022). HMGB1 in nervous system diseases: A common biomarker and potential therapeutic target. Front. Neurol..

[31] Liang K., Li X., Guo Q., Ma J., Yang H., Fan Y., Yang D., Shi X., She Z., Qi X. (2024). Structural changes in the retina and serum HMGB1 levels are associated with decreased cognitive function in patients with Parkinson’s disease. Neurobiol. Dis..

